# Seoul Orthohantavirus in Wild Black Rats, Senegal, 2012–2013

**DOI:** 10.3201/eid2610.201306

**Published:** 2020-10

**Authors:** Moussa M. Diagne, Idrissa Dieng, Laurent Granjon, Héloïse Lucaccioni, Abdourahmane Sow, Oumar Ndiaye, Martin Faye, Khalilou Bâ, Yamar Bâ, Mamoudou Diallo, Oumar Faye, Jean-Marc Duplantier, Mawlouth Diallo, Pascal Handschumacher, Ousmane Faye, Amadou A. Sall

**Affiliations:** Institut Pasteur, Dakar, Senegal (M.M. Diagne, I. Dieng, A. Sow, O. Ndiaye, M. Faye, Y. Bâ, Oum. Faye, Maw. Diallo, Ous. Faye, A.A. Sall);; Centre de Gestion des Populations, Institut de Recherche pour le Développement, Montpellier, France (L. Granjon, J.-M. Duplantier);; Université Paris Nanterre, Nanterre, France (H. Lucaccioni);; Institut de recherche pour le développement Senegal, Dakar (K. Bâ, Mam. Diallo);; Sciences Economiques & Sociales de la Santé & Traitement de l’Information Médicale, Marseille, France (P. Handschumacher);; Aix Marseille University, Marseille (P. Handschumacher);; Institut National de la Santé et de la Recherche Médicale, Marseille (P. Handschumacher)

**Keywords:** Hantavirus, Seoul orthohantavirus, rodents, black rats, Senegal, viruses, zoonoses, Rattus rattus

## Abstract

Hantaviruses cause hemorrhagic fever in humans worldwide. However, few hantavirus surveillance campaigns occur in Africa. We detected Seoul orthohantavirus in black rats in Senegal, although we did not find serologic evidence of this disease in humans. These findings highlight the need for increased surveillance of hantaviruses in this region.

Hantaviruses (family *Hantaviridae*, genus *Orthohantavirus*) are RNA viruses transmitted by aerosolized excreta from infected rodents and shrews. In humans, they cause hemorrhagic fever with renal syndrome (more often observed in Asia and Europe) and cardiopulmonary syndrome (more common in the Americas) (*1*). Only 1 case has been confirmed in Africa, in the Central African Republic in 1987 (*2*). However, studies from 2006 through 2013 have discovered new hantaviruses in autochthonous African rodents, moles, and bats (*3*,*4*). In addition, serologic evidence in humans and rodents in Africa suggest local circulation (*5*). For example, a study in rural areas of Senegal found 11.5% of rodents and 16.6% of humans had antibodies against hantaviruses (*3*). More recently, serologic evidence of hantaviruses was reported in domestic and peridomestic rodents from some regions in Senegal (*6*).

Southeastern Senegal has become a major trade area because of urbanization and substantial improvement of its road and rail networks in the late 1990s (*7*). Within a few years, these changes led to the rapid spread of a major invasive rodent species, the black rat (*Rattus rattus* [family *Murinae*]), which is a reservoir for Seoul orthohantavirus (SEOV) (*4*,*5*,*7*). To assess the prevalence of hantaviruses in rodents, we screened for hantaviruses in *R. rattus* rats and commensal or peridomestic co-existing rodents in 2012–2013, approximately 15 years after the 1998 opening of a tarred road in eastern Senegal.

## The Study

The national ethics committee for research of Senegal approved the study (authorization no. 0360-MSAS/DPRS/DR, on October 24, 2011). During May 2012–December 2013, we trapped small mammals as previously described (*8*) inside dwelling places and their surroundings (immediate and local) over periods of 1–6 consecutive days.

We caught 1,414 small mammals, including 403 black rats, from 10 different species (Appendix Table). We sampled whole blood, brain, and visceral organ tissues, which we then transferred to the Institut Pasteur (Dakar, Senegal). We triturated each solid sample in Leibovitz-15 medium (GIBCO-BRL, https://www.thermofisher.com) and centrifuged them to collect the suspension. To collect serum, we centrifuged whole blood samples. We extracted RNA from these different suspensions using the QIAamp RNA Viral Kit (QIAGEN, https://www.qiagen.com) according to the manufacturer’s recommendations. To make cDNA, we used avian myeloblastosis virus reverse transcriptase (Promega, https://www.promega.com) followed by a nested conventional PCR with GoTaq Polymerase (Promega, https://www.promega.com) and a highly conserved hantavirus primers system selective for the partial large segment protein gene (*9*). We sequenced amplicons using GENEWIZ (https://www.genewiz.com), assembled them using EMBOSS Merger software (http://www.bioinformatics.nl/cgi-bin/emboss/merger), and analyzed them with BLAST (http://blast.ncbi.nlm.nih.gov/Blast.cgi). We performed sequence alignment with Mafft (*10*) and built a maximum-likelihood phylogenetic tree with iQ-TREE (*11*), using 1,000 replicates for bootstrapping.

**Figure 1:**
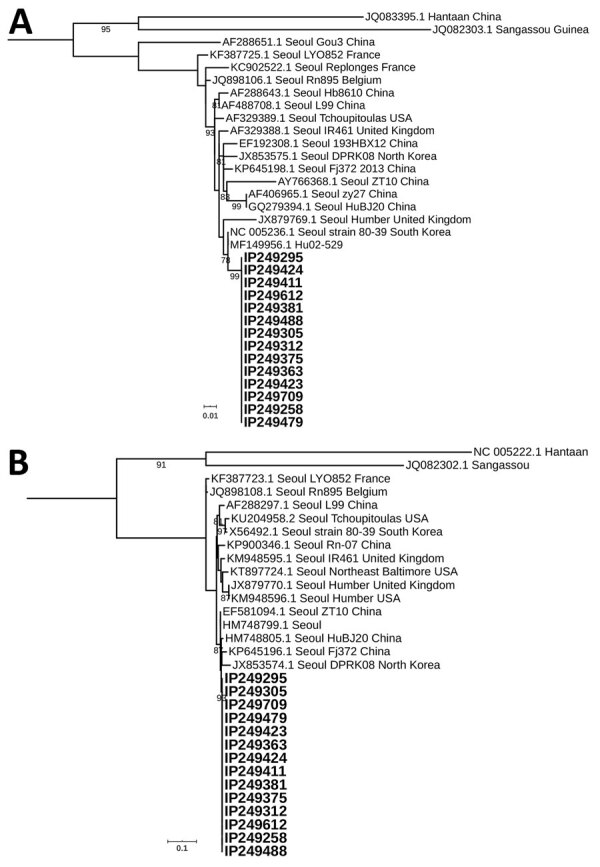
Phylogenetic analysis of Seoul orthohantavirus strains from black rats (*Rattus rattus* [family *Murinae*]; boldface) and reference sequences, Senegal, 2012–2013. Phylogenetic trees were generated by the maximum-likelihood method using the transition plus invariate sites plus gamma 4 model of the small segment (266 nt) (A) and the large segment (347 nt) (B). The numbers at each node are bootstrap probabilities (>90%) as determined for 1,000 iterations. GenBank numbers are indicated for reference sequences. Scale bars indicate 0.01 substitutions per nucleotide (A) and 0.1 substitutions per nucleotide (B).

Of the 1,414 mammals, 13 black rats tested positive for hantavirus RNA. We detected RNA in 14 samples: 9 brain homogenates, 4 multiorgan homogenates, and 1 serum sample. We confirmed the positive samples using PCR with highly conserved hantavirus small segment primers (*12*). Sequence analysis of partial large (deposited under GenBank accession nos. MT276868–81) and small (deposited under GenBank accession nos. MT276854–67) segments revealed 99.42% identity with SEOV strain Rn-HD27 from China (GenBank accession no. HM748799) and 99.64% identity with SEOV strain Hu02-529 from South Korea (GenBank accession no. MF149956) (Figure 1).

**Figure 2:**
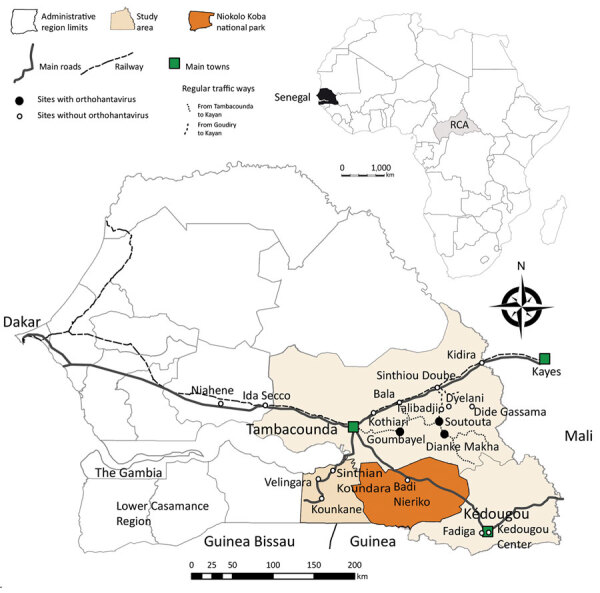
Locations of trapping sites (circles) used in study of rodentborne Seoul orthohantavirus in Senegal, 2012–2013. Black circles indicate trapping locations of Seoul orthohantavirus–infected black rats (*Rattus rattus* [family *Murinae*]). Inset shows location of Senegal in Africa. Map created using the package maptools installed in R studio version 1.2.1335 (https://rstudio.com/products/rstudio/) and shapefiles downloaded from the free domain of the Geographic Information System (http://www.diva-gis.org).

We detected SEOV RNA in 13 black rats caught in 3 villages: Goumbayel (7 rodents), Soutouta (4 rodents), and Dianke Makha (2 rodents). These villages were located »1 hour’s drive from the main road between Tambacounda and Kidira (Figure 2). Frequent movement of goods and humans between these 3 villages might explain the low genetic diversity among the new SEOV strains from black rats. We did not observe signs of disease in the infected animals at the time of capture. Of the 4 villages that yielded the highest numbers of black rats in this study, 3 harbored rats infected with SEOV (Figure 2) (*7*). High densities of black rats might contribute to the occurrence of hantavirus in these villages, especially because host demography might affect hantavirus circulation (*13*).

Seasonal patterns might complicate these findings. We surveyed the villages harboring SEOV-infected rats in February 2013, which might be a favorable period for rodent reproduction, population increase, and thus hantavirus circulation (*13*). Despite the presence of juveniles, *R. rattus* populations had relatively high proportions of sexually active animals (75% in Goumbayel, 48% in Soutouta, and 71% in Dianke Makha) (Appendix Figure). These data suggest that high level of interactions (male–female, adult–juvenile) occurred in these populations during that period, possibly promoting viral circulation. Conversely, we investigated nearby villages (Dieylani, Dide Gassama, Koussan, and Talibadji, in which we did not find evidence of hantavirus-infected black rats) in October 2012, at the end of the rainy season. Our investigations in May 2012 and November 2013 of the Kedougou area did not detect evidence of SEOV.

To assess potential human transmission, we performed parallel studies of human populations in some villages. Participants consented to an interview about rodent exposure and gave blood samples. During October 2012–March 2013, we recruited 541 participants with a mean age of 24 years (range 2–91 years) (Table). Of the 541 participants, 372 (68.8%) reported close contact with rodents. The highest rates of rodent exposure were in Soutouta and Sinthiou Doube (Table). We performed an in-house ELISA specific to IgG against SEOV on the human serum samples using reagents from the US Centers for Disease Control and Prevention (Atlanta, GA, USA). No IgG against SEOV was detected in the tested human samples, regardless of whether the participant’s village had evidence of SEOV-infected black rats; this finding suggests a lack of human exposure. The role of species diversity in virus transmission is extremely complex (*14*). The relatively low SEOV prevalence in black rats (Appendix Table) might explain the negative results of the human serologic survey.

**Table:** Human exposures to rodents in selected villages, Senegal, 2012–2013

Village/town	No. participants	No. (%) participants in contact with rodents	No. distinct species encountered	Black rats	Time period
Tambacounda					
Youpe Hamady	87	70 (80.5)	4	No	2012 Oct 19–20
Talibadji	33	11 (33.3)	3	Yes	2012 Oct 21
Sinthiou Doube	39	37 (94.9)	4	Yes	2012 Oct 22
Ndiobene	45	20 (44.4)	2	No	2012 Oct 22
Dianke Makha	101	40 (39.6)	5	Yes	2012 Sep 10
Soutouta	89	83 (93.3)	4	Yes	2012 Sep 11
Kedougou					
Kedougou	147	111 (75.5)	6	Yes	2013 Mar 9–10
Total	541	372 (68.8)			

## Conclusions

We found SEOV, a hantavirus pathogenic to humans, in black rats in southeastern Senegal. Phylogenic analyses grouped the newly detected SEOV with strains from Asia. Exchanges between Africa and Asia can potentially increase the opportunities for pathogens to expand their geographic range as previously described (*15*).

In-depth phylogenetic analysis of complete genomes would help elucidate the molecular evolution of this virus in Africa. This study highlights the need to improve hantavirus surveillance in Senegal and other countries in Africa for public health prevention strategies.

Appendix Additional information on detection of Seoul orthohantavirus in wild black rats, Senegal
