# Supplementary material for: Seoul Orthohantavirus in Wild Black Rats, Senegal, 2012–2013
Source: Emerg Infect Dis. 2020 Oct;26(10):2460–4. doi: 10.3201/eid2610.201306 (PMC7510722; doi:10.3201/eid2610.201306)
Supplement: Appendix — Additional information on detection of Seoul orthohantavirus in wild black rats, Senegal [file 20-1306-Techapp-s1.pdf]

Article DOI: https://doi.org/10.3201/eid2610.201306

Seoul Orthohantavirus in Wild Black Rats, Senegal, 2012–2013

Appendix

Appendix Table. Small mammals trapped for Hantavirus screening and *Seoul Orthohantavirus* prevalence among black rats, Senegal, 2012–2013

Localities studied	The African grass rat (*Arvicanthis niloticus*) (no.)	The Gambian pouched rat (*Cricetomys gambianus*) (no.)	African giant shrew (*Crocidura olivieri*) (no.)	The Gambian gerbil (*Gerbilliscus gambianus*) (no.)	The Guinea multimammate mouse (*Mastomys erythroleucus*) (no.)	The Natal multimammate mouse (*Mastomys natalensis)* (no.)	The house mouse (*Mus musculus*) (no.)	Dalton's mouse (*Praomys daltoni*) (no.)	Fat mouse (*Steatomys sp*) (no.)	Black rat (*Rattus rattus*) (no.)	Seoul virus prevalence in black rats (%)
Bala	9	0	32	0	6	0	6	1	0	7	0
Bedi Nieriko	0	0	6	0	14	0	0	0	0	25	0
Dianke Makha	13	0	10	0	14	0	0	4	0	33	6.1
Dide Gassama	0	0	0	0	0	0	0	0	0	30	0
Dyelani	0	0	3	0	1	0	0	2	0	12	0
Fadiga	0	0	3	0	0	0	0	0	0	0	0
Goumbayel	0	0	20	0	7	0	0	1	0	36	19.4
Ida Seco	9	0	0	1	4	0	14	0	1	8	0
Kedougou center	4	1	18	0	4	241	0	6	0	74	0
Kidira	11	0	11	0	2	0	16	12	0	3	0
Kothiari	0	0	20	0	2	0	22	0	0	20	0
Kounkane	0	0	1	0	2	0	11	2	0	10	0
Niahene	1	0	28	0	3	0	83	0	1	13	0
Sinthian Koundara	0	0	16	0	2	0	0	2	0	28	0
Sinthiou Doumbe	0	0	4	0	7	0	0	1	0	18	0
Soutouta	14	0	14	0	7	0	0	0	0	21	19.1
Talibadji	0	0	22	0	8	0	0	0	0	2	0
Tambacounda	18	0	65	0	11	0	73	21	0	38	0
Velingara	0	0	21	0	0	0	22	0	0	25	0
Total	79	1	294	1	94	241	247	52	2	403	3.2

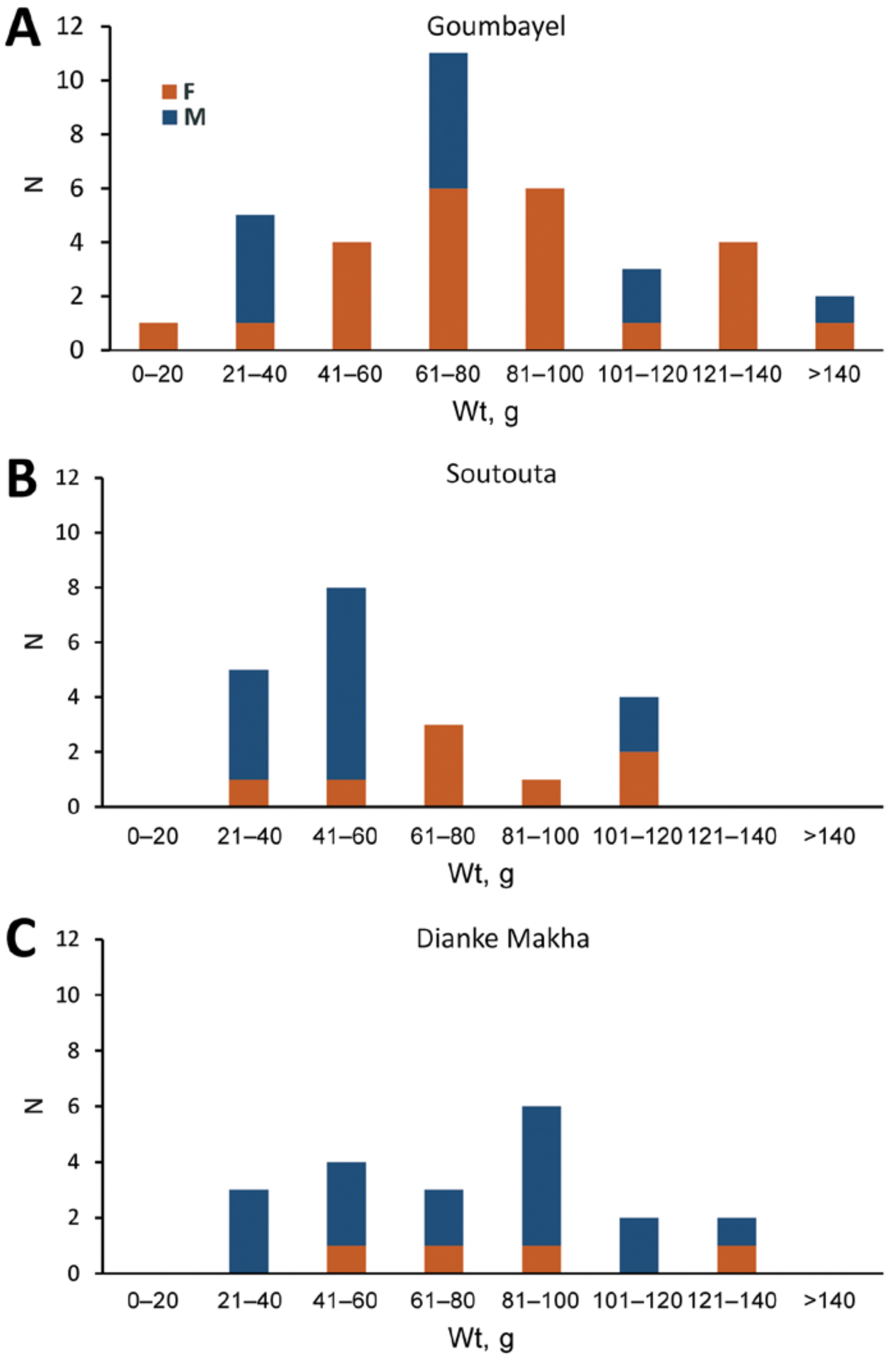

Appendix Figure. Population distributions of black rats trapped in: (A) Goumbayel, where 19 (79%) of 24 females were sexually active, 8 (67%) of 12 males were sexually active, and 6 (17%) of 36 individuals were juveniles; (B) Soutouta, where 3 (37.5%) of 8 females were sexually active, 7 (54%) of 13 males were sexually active, and 8 (38%) of 21 individuals were juveniles; and (C) Dianke Makha, where 2 (40%) of 5 females were sexually active, 13 (81%) of 16 males were sexually active, and 6 (29%) of 21 individuals were juveniles, Senegal, 2012–2013.
